# Prevalence of severe asthma according to the drug regulatory agency perspective: An Italian experience

**DOI:** 10.1016/j.waojou.2019.100032

**Published:** 2019-05-10

**Authors:** A. Vianello, M. Caminati, M. Andretta, A.M. Menti, S. Tognella, G. Senna, L. Degli Esposti

**Affiliations:** Respiratory Pathophysiology Division, University-City Hospital of Padua, Padua, Italy; Asthma Center and Allergy Unit, Verona University and General Hospital, Verona, Italy; Department of Medicine, University of Verona, Verona, Italy; Health Technology Assessment Unit, Azienda Zero, Padova, Italy; Respiratory Unit, Orlandi General Hospital, Bussolengo, Verona, Italy; Asthma Center and Allergy Unit, Verona University and General Hospital, Verona, Italy; CliCon S.r.l. Health, Economics & Outcomes Research, Ravenna, Italy

**Keywords:** Asthma, Severe asthma, Asthma prevalence, Treatment adherence

Severe asthma prevalence is still controversial.^1^ Although the ERS/ATS (European Respiratory Society / American Thoracic Society) guidelines have recently established a shared definition of severe asthma,^2^ the range of its frequency reported by the few published epidemiological studies is impressively large (1.8–38%).^1^ Differences in population samples, definitions and methodology, together with the complexity of this sort of research, may account for that variability.^2^, ^3^, ^4^, ^5^

The present study aimed at investigating the prevalence of severe and uncontrolled severe asthma in the general population through the analysis of the electronic database of Veneto region (North East of Italy) Drug Regulatory Agency.

A 5-years retrospective (2011–2016) cross-sectional analysis was performed, including subjects older than 6. The database provides the information regarding the anti-asthmatic drugs prescriptions and the asthma-related hospitalizations for all the people living in the region and referring to the National Health System (NHS) services.

The ERS/ATS definition^2^ and the GINA recommendations^6^ were considered for the severity assessment of asthmatic patients, who were identified as follows (Fig. 1):

- The whole asthmatic population included subjects with the exemption code (007), which is released by the NHS to asthmatic patients after specialist's certification. Usually, but not always, the certification implies the lung function assessment. Subject with the exemption code, with or without spirometry assessment, were considered. Patients with Chronic Obstructive Pulmonary Disease (COPD), identified by a different exemption code (057), although prescribed with the same drugs (ICS/LABA combination), were excluded. As per Italian national health system regulation, the two exemption codes are mutually exclusive.
- Among asthmatic patients, the more severe ones were selected on the basis of anti-asthmatic treatment level in accordance with step 4 and 5 GINA recommendations (ICS/LABA combination plus another controller drug, i.e. antileukotrienes, theophylline, tiotropium).^6^ The subgroup of patients experiencing more than 2 exacerbations/year (hospitalization or use of oral corticosteroid (OCS) longer than three days) was also identified.
- Within the severe asthma population, patients adherent to the treatment were identified. Adherence was considered as a use over 80% of the prescribed treatment within a 12-months period, that is the amount of drugs bought by the patients in one year. Patients who, despite a regular GINA step 4–5 treatment had more than 2 exacerbations/year, needed hospitalization, or used OCS on regular basis for 6 months, were defined as uncontrolled.

**Fig. 1:**
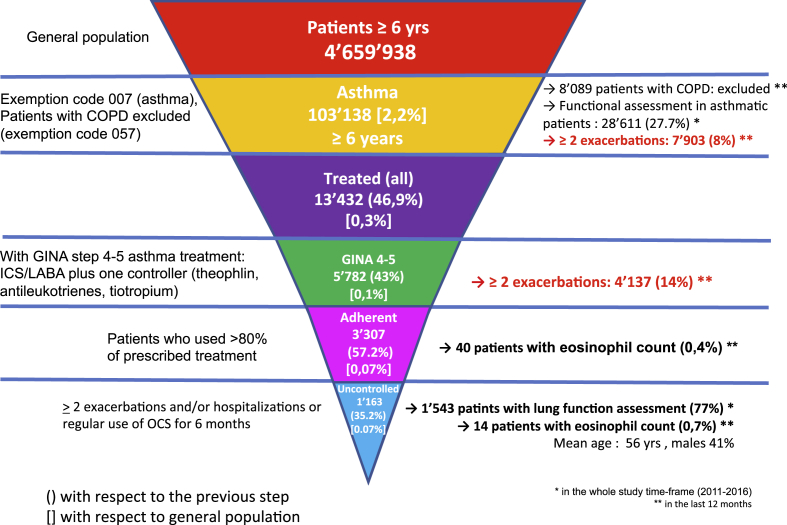
Prevalence trends of different asthma severity clusters.

The database included 4.659.938 subjects (general population). The overall asthma prevalence was 2.2% (103.138 subjects) (Fig. 1). A respiratory assessment was performed in 28.611 (27,7%) of them. In the asthma population, 8% of patients suffered from more than 2 exacerbations/year. According to the treatment, 43% of treated asthmatics (0.1% of general population) belonged to GINA steps 4 and 5, 291 of them (5.0%) assuming omalizumab treatment (it was the only biologic available in the area where the study was conducted at the time of the analysis). Within the same subpopulation, 57.2% (0.1% of general population) were adherent to the treatment.

Among adherent patients, 1.163 (35.2% of severe asthmatics and 0.1% of general population) remained uncontrolled. In this last subgroup, in the whole study time frame (2011–2016) a spirometry was performed in 77% of cases and considering the 12 months prior to the inclusion in the cross-sectional analysis the eosinophil count was available in 0.7% of patients. The mean age of severe uncontrolled asthmatics was 56 years and females were predominant (59%). According to these data, based on prescribed and dispensed asthma medications, the prevalence of severe asthma is 0.07% in the general population and 3.2% among asthmatic patients. Patients uncontrolled despite a regular GINA step 4/5 treatment were 1.2% of asthmatics. In order to obtain a more reliable estimate, only adherent patients were considered for defining severe asthma and severe uncontrolled asthma population.

The functional assessment was performed in 27.7% of patients with certified asthma and among patients with uncontrolled severe disease 23% did not perform a spirometry.

To the best of our knowledge this is the first investigation on severe and severe uncontrolled asthma prevalence in Italy, reporting data from a general population database. Only patients over 6 years were included, as the prevalence of severe asthma in Youngers is negligible.^7^ In accordance with previous studies severe asthma seems to be more frequent in females and in adults.^1^, ^2^, ^3^, ^4^, ^5^ Although similarities in the study methods, our findings are not completely in accordance with a Danish cross-sectional study analyzing a nationwide prescription database and reporting that, on the basis of antiasthma treatment level, 8.1% of patients suffered from severe asthma.^3^ Similarly, as a result of an electronic database analysis in Israel, the prevalence of severe asthma was estimated around 5% of all asthmatics, classified on the basis of medications dispensed.^5^ The geographical variability may account for it together with, in our case, the wider age range of the population included. Furthermore, in the Danish study,^3^ the severe asthma group included only patients treated with high dose ICS/LABA combination plus another controller (i.e. LABA, Xanthines or LTRA) or with omalizumab, whilst in our analysis patients under GINA steps 4 and 5 treatment, meaning medium or high dose ICS/LABA combination plus another controller were labeled as severe. Also, in our study only patients adherent to the treatment were considered for estimating severe asthma prevalence.

However, differently from the other reports, our analysis relied on a general population database provided by the regional Drug Regulatory Agency, which can probably be considered more accurate under the epidemiological perspective. Another study performed in The Netherlands estimated that 3.6% of asthmatics is affected by severe refractory disease. The diagnosis of asthma and degree of asthma control were derived from patient-self-completed questionnaires.^4^ In this case the risk of bias is even higher. In the Israel study^5^ about 30% of severe asthmatics were uncontrolled, due to emergency room visits and hospitalizations for asthma exacerbations. According to our findings, 20% of severe asthmatic patients remained uncontrolled. Differently from the Israel study, in our analysis uncontrolled severe asthma was considered only once the adherence to the treatment was verified, as previously described. Also, in our dataset severe uncontrolled patients were defined when experiencing more than 2 exacerbations/year, needing hospitalization, or using OCS on regular basis for 6 months. The last is considered by the ERS/ATS guidelines as a criterion for defining severe asthma and not its control. This difference, although considered by the authors an acceptable proxy definition for the purpose of this study, should be taken into consideration when comparing our results with other reports.

In accordance with previous studies the lung function assessment was not regularly performed.^1^ Of note 23% of subjects with severe uncontrolled disease did not undertake it in the last five years. The evaluation of the blood eosinophil count is still uncommon despite its relevance both for the phenotype identification and for the biologic treatments selection. Blood eosinophilia also represents a marker of response to corticosteroid treatment and a predictor of exacerbations.^2^

Some limitations have to be highlighted. First, the database excluded the patients referring to private practice and/or without the exemption code. However, especially in the field of severe asthma, due to the costs of drugs and the complex management it is reasonable to consider that most of patients refer to the NHS services, so that this limitation should not represent a substantial bias. In fact, in the case the patients are followed-up in the context of the NHS, it implies that everything concerning the management of their disease, namely exams and treatments, are accurately registered. Second, the method used for adherence assessment may not exactly reflect the amount of really taken medications. However among the available tool for adherence evaluation, no one is completely free from bias. Furthermore, the applied method can be a way to distinguish true difficult to treat asthma and lack of asthma control due to poor treatment adherence.

As severe and severe uncontrolled asthma prevalence assessment is crucial for disease management, prevention strategies and cost/benefit estimations,^1^, ^8^ further and larger studies at a national and international level are needed.

## Declarations

### Authors’ contributions

AV, GS, MC and MA conceived the article structure and drafted the manuscript. LDE analyzed the dataset. All the authors critically revised the manuscript draft and approved the final manuscript.

### Ethics approval and consent to participate

Ethics approval was obtained from the Health Technology Assessment Unit Committee.

### Consent for publication

Not applicable.

### Availability of data and materials

Data sharing will be provided following a request to the corresponding author.

### Competing interests

The authors declare that they have no competing interests.

### Funding

Not applicable.
